# Perceptions of Undergraduate Medical Students Toward Integrated Management of Childhood Illness (IMCI) Pre-service Education at Sultan Qaboos University, Muscat

**DOI:** 10.7759/cureus.47260

**Published:** 2023-10-18

**Authors:** Mohammed Al-Yahyahi, Maisa Al Kiyumi, Sanjay Jaju, Muna Al Saadoon

**Affiliations:** 1 Family Medicine, College of Medicine and Health Sciences, Sultan Qaboos University, Muscat, OMN; 2 Family Medicine and Public Health, College of Medicine and Health Sciences, Sultan Qaboos University, Muscat, OMN; 3 Child Health, College of Medicine and Health Sciences, Sultan Qaboos University, Muscat, OMN

**Keywords:** oman, sultan qaboos university (squ), childhood illnesses, imci, students perceptions

## Abstract

Background

Inconsistent evidence concerning the clinical practice implications of the Integrated Management of Childhood Illness (IMCI) pre-service education exists in the literature. The aim of this study is to assess the IMCI pre-service training perceptions of medical students, including their willingness to prospectively utilize the IMCI guidelines in clinical settings.

Methods

This is an observational cross-sectional study that was conducted between June 1 and August 31, 2022, at the College of Medicine and Health Sciences, Sultan Qaboos University (SQU), Muscat, Sultanate of Oman. The demographic data and IMCI pre-service education perceptions were recorded via the 10 close-ended questions. The questions focused on the student’s perception of the usefulness of IMCI pre-service training in improving their knowledge, attitude, and practice (KAP) regarding childhood illnesses and how well it has enhanced their skills in dealing with sick children. SPSS Statistics version 26.0 (IBM Corp. Released 2019. IBM SPSS Statistics for Windows, Version 26.0. Armonk, NY: IBM Corp.) was used to analyze the data.

Results

A total of 196 responses were collected, with 117 of them being from female participants and the remaining 79 from male participants. Participants were subcategorized into phase 2 (n=103), phase 3A (pre-clerkship, n=45), and phase 3B (junior clerkship, n=48). At least 67.8% of 171 medical students complying with one to two training sessions intended to apply their IMCI pre-service education knowledge and skills in clinical practice and parental counseling. The medical knowledge and clinical practice skill enhancement abilities of the IMCI sessions were recognized by ≥49.7% of medical students. The student responses regarding childhood illness management (p=0.03) and holistic assessment confidence (p=0.042) varied significantly between the study phases. The IMCI pre-service skills, knowledge, and confidence levels were observed in 47.1% (phase 2), 13.2% (phase 3A), and 35.5% (phase 3B) of medical students. Similarly, 40.2% (phase 2), 23.7% (phase 3A), and 54.8% (phase 3B) of subjects believed in the IMCI pre-service training’s influence on their ability to perform holistic assessments in the pediatric population.

Conclusion

The overall results of this study advocate the clinical practice implications, based on the positive student perceptions, of the IMCI pre-service training in SQU. Future qualitative studies should evaluate these findings with wider student populations.

## Introduction

The global mortality rate in children (<5 years of age) was reported to be five million in 2020, corresponding to approximately 13,800 deaths per day [1]. A high prevalence of child mortality was observed in middle- and low-income nations, and the leading causes of such deaths included malaria, diarrhea, pneumonia, and other preventable infections [1]. The Integrated Management of Childhood Illness (IMCI) approach was developed by the United Nations International Children’s Emergency Fund (UNICEF) and World Health Organization (WHO) at the beginning of the 1990s [2]. The IMCI strategy investigated the potential causes of child mortality by upskilling healthcare professionals and improving the overall quality of patient management practices [3]. More than 100 nations to date have adopted IMCI since its inception in 1996 [4]. The child health improvement potential of the IMCI has been examined periodically via multifactorial assessments. However, the high incidence of childhood mortality across the globe, despite IMCI implementation, warrants the reassessment of this approach and its implementation paradigm through evidence-based research [4]. The latest evidence, however, indicates the non-compliance of healthcare professionals with the IMCI guidelines in clinical settings [5]. Interestingly, most physicians continue to trust anecdotal evidence and their own patient management skills while ignoring the IMCI recommendations. This might eventually add to the rising child mortality rates on a global scale [6,7].

Alternatively, many countries continued to witness the benefits of IMCI implementation in terms of improving children’s healthcare quality and treatment outcomes [8]. Importantly, a 13% decline in pediatric death rates was observed in Tanzania in 2005 [9]. Additionally, a range of studies conducted between 2005 and 2010 revealed substantial enhancements in healthcare quality and reductions in treatment costs and child mortality rates, following the IMCI implementation in pediatric care settings [10-14]. The reported barriers to IMCI implementation included physician resistance, prolonged consultations, long-term training sessions, and customized healthcare policies [15,16]. In 1999, IMCI was officially introduced into the healthcare system in Oman. In addition, several studies investigated predominant attributes, including patient-physician communication and antibiotic administration guidelines [6,7]. Indeed, there is a considerable amount of evidence regarding the positive role of IMCI pre-service education in transforming pediatric healthcare outcomes, including mortality rates. A recent case-control study indicated the positive impact of the IMCI pre-service education on the disease assessment and patient management potential of medical students [17]. Evidence also demonstrates the clinical practice implications of IMCI pre-service education based on its ability to improve the practical and theoretical knowledge of medical students [6,7].

At Sultan Qaboos University (SQU), the curriculum of medical degree MD is divided into three phases. Phase 1 begins in the first year of the program, focusing on foundational knowledge. Phase 2 encompasses both the second and third years, providing a comprehensive academic experience. Phase 3A corresponds to the pre-clerkship phase, commencing at the beginning of the fourth year and emphasizing clinical readiness. Finally, phase 3B covers both junior and senior clerkships and extends through the fourth, fifth, and sixth years, allowing students to gain extensive practical experience in clinical settings. These phases provide a structured path for students as they progress through their medical education.

The IMCI was incorporated into the curriculum of undergraduate medical students in 2007. It was integrated into lectures during the second phase (phase 2). These lectures were designed to familiarize students with IMCI, its components, common childhood disorders, and treatment concepts. Students have additional opportunities to practice the IMCI strategy in phase 3 through their placements at primary healthcare facilities. Additionally, the IMCI was incorporated into the summative evaluations for the rotations. The SQU pre-service IMCI training, however, is not supported by substantial evidence. Accordingly, the present study aims to investigate SQU medical students' perceptions of IMCI pre-service education. The outcomes of this assessment will help understand the IMCI pre-service training perceptions of medical students and their ability to utilize its outcomes as prospective healthcare providers. Importantly, the results of this study will help establish the required milestones warranted to improve IMCI pre-service education and its potential outcomes.

## Materials and methods

Study design

This is a cross-sectional study that was conducted in SQU’s College of Medicine and Health Sciences, Muscat, from June 1 to August 30, 2022. All students from phase 2 and phase 3 (3A: pre-clerkship and 3B: junior clerkship), who have attended at least one session on IMCI, were included in this study. Exclusion criteria included all phase 1 students, students who had never attended any IMCI sessions, and those who declined to participate. All eligible students were invited to participate and the purpose of the study was explained to them by the co-investigators. An informed written consent was obtained from those who agreed to participate. Furthermore, the process was anonymous in order to maintain the confidentiality of the students, and the enrolled participants at each phase (phases 2, 3A, and 3B) were assigned a unique number in chronological order. The Medical Research Ethics Committee issued approval SQU-EC/136/2022MREC#2757.

Questionnaire

The eligible participants were asked to fill in a self-administered questionnaire. The questionnaire consisted of two parts. Part one is the sociodemographic characteristics, such as age, gender, attending IMCI lectures, and the level of training. The second part consisted of ten multiple-choice questions related to the perception of undergraduate medical students toward IMCI training. The students' impressions of IMCI training and their desire to employ this training and skills in future practice were assessed using a reliable and well-validated structured questionnaire containing six constructs: (i) “perceived usefulness,” (ii) “enhanced assessment skills,” (iii) “enhanced knowledge,” (iv) “attitudes and skills (KAS),” (v) enhanced confidence,” and (vi) “enhanced counseling skills and future intention to use IMCI in a clinical setting” (19). Permission to use the questionnaire was obtained from the corresponding author (19).

Sample size

The sample size was determined for a finite population in each phase of the study, using the Krejcie and Morgan table [18], as shown in Table 1. Investigators utilized a consecutive sampling approach to recruit the study subjects.

**Table 1:** Sample size and response rates among different groups

Student category	Finite population size, n	Required sample size, n	The sample consent for the study, n	Response rate, %
Phase 2	266	71	103	>100
Phase 3A (pre-clerkship)	126	54	45	83
Phase 3B (junior clerkship)	135	56	48	86

Statistical analysis

SPSS Statistics version 26.0 (IBM Corp. Released 2019. IBM SPSS Statistics for Windows, Version 26.0. Armonk, NY: IBM Corp.) was used to analyze the inputs from the participants. Frequency tables were used for the descriptive analysis. The chi-squared test was used to analyze the variance between the responses of the participants from different phases. A p-value of <0.05 was considered significant.

## Results

A total of 196 responses were obtained, 117 of which were from females and the remaining 79 from males (female-to-male ratio: 1.5:1). The ages of the participants ranged between 20 and 24 years. There were 103 responses from students from phase 2, compared to 93 from phase 3 (45 and 48 from phases 3A and 3B, respectively). Table 1 depicts the sample size and response rates observed during phase 2 and phase 3 (A&B) of the study. Most students (129) had two lectures/tutorials, followed by 42 students who had only one lecture/tutorial. The distribution of the students according to the duration of pre-service IMCI training they have received is illustrated in Table 2. The analysis was based on the categorization of students according to the duration of pre-service IMCI training they had received. Data from the last three student categories were examined by considering them as one independent variable.

**Table 2:** Distribution of the undergraduate medical students according to the duration of pre-service IMCI training they have received IMCI: Integrated Management of Childhood Illness

Educational level	Duration of pre-service IMCI training received					Total
	One lecture/tutorial	Two lectures/tutorials	≥3 lectures/tutorials	Practical sessions	Both lectures and practical sessions	
Phase 2	11	91	1	0	0	103
Phase 3A (pre-clerkship)	17	21	6	1	0	45
Phase 3B (junior clerkship)	14	17	2	12	3	48
Total	42	129	9	13	3	196

Varied responses were obtained from medical students attending one to two IMCI tutorials/lectures (Table 3). The IMCI training effectiveness was acknowledged by the majority of students who participated in phase 2 (79.4%), phase 3A (76.3%), and phase 3B (54.8%) of the study. Contrarily, 14.7% of phase 2, 21.1% of phase 3A, and 32.3% of phase 3B students had no insight into the clinical practice implications of the IMCI approach. Disagreement against the benefits of IMCI training was registered by a minority of the students across the three phases (6.4%).

**Table 3:** Responses obtained from undergraduate medical students attending one to two IMCI tutorials/lectures IMCI: Integrated Management of Childhood Illness

Participants (n=171)		Educational level			Total	p-value
		Phase 2 n(%)	Phase 3A (pre-clerkship) n(%)	Phase 3B (junior clerkship) n(%)		
IMCI training/orientation is an effective teaching method.	Agree	81 (79.4%)	29 (67.3%)	17 (54.8%)	127 (74.3%)	>0.05
	Disagree	6 (5.9%)	1 (2.6%)	4 (12.9%)	11 (6.4%)	
	I don't know	15 (14.7%)	8 (21.1%)	10 (32.3%)	33 (19.3%)	
Total		102 (100%)	38 (100%)	31 (100%)	171 (100%)	
Pre-service IMCI — training has enhanced my medical skills.	Agree	56 (54.9%)	17 (44.7%)	13 (41.9%)	86 (50.3%)	>0.05
	Disagree	8 (7.8%)	6 (15.8%)	8 (25.8%)	22 (12.9%)	
	I don't know	38 (37.3%)	15 (39.5%)	10 (32.3%)	63 (36.8%)	
Total		102 (100%)	38 (100%)	31(100%)	171(100%)	
Pre-service IMCI — training is a practical approach for improved Assessment of childhood illnesses.	Agree	75 (73.5%)	22 (57.9%)	18(58.1%)	115 (67.3%)	>0.05
	Disagree	5 (4.9%)	1(2.6%)	3 (9.7%)	9 (5.3%)	
	I don't know	22 (21.6%)	15 (39.5%)	10 (32.3%)	47 (27.5%)	
Total		102 (100%)	38 (100%)	31 (100%)	171 (100%)	
Pre-service IMCI — training has improved my clinical Knowledge.	Agree	55 (53.9%)	18 (47.4%)	12 (38.7%)	85 (49.7%)	>0.05
	Disagree	11 (10.8%)	4 (10.5%)	3 (9.7%)	18 (10.5%)	
	I don't know	36 (35.5%)	16 (42.1%)	16 (51.6%)	68 (39.8%)	
Total		102 (100%)	38 (100%)	31 (100%)	171 (100%)	
Because of pre-service IMCI —training, I feel confident in dealing with sick children.	Agree	48 (47%)	5(13.2%)	11 (35.5%)	64 (37.4%)	0.003
	Disagree	22 (21.6%)	9(23.7%)	8(25.8%)	39 (22.8%)	
	I don't know	32 (31.4%)	24 (63.2%)	12 (39.7%)	68 (39.8%)	
Total		102 (100%)	38 (100%)	31 (100%)	171 (100%)	
Pre-service IMCI — training has improved my counseling skills.	Agree	45 (44.1%)	9 (23.7.%)	9 (29.0%)	63 (36.8%)	>0.05
	Disagree	16 (15.7%)	9 (23.7%)	9 (29.0%)	34 (19.9%)	
	I don't know	41 (40.2%)	20 (52.6%)	13 (41.9%)	74 (43.3%)	
Total		102 (100%)	38 (100%)	31 (100%)	171 (100%)	
Pre-service IMCI — training has helped me to assess a child holistically.	Agree	41 (40.2%)	9 (23.7%)	17 (54.8%)	67 (39.2%)	0.042
	Disagree	15 (14.7%)	9 (23.7%)	7 (22.6%)	31 (18.1%)	
	I don't know	46 (45.1%)	20 (52.6%)	7 (22.6%)	73 (42.7%)	
Total		102 (100%)	38 (100%)	31 (100%)	171 (100%)	
All in all, pre-service IMCI — training is an effective teaching approach.	Agree	73 (71.6%)	21 (55.3%)	19 (61.3%)	113 (66.1%)	>0.05
	Disagree	8 (7.8%)	1 (2.6%)	4 (12.9%)	13 (7.6%)	
	I don't know	21 (20.6%)	16 (42.1%)	8(25.8%)	45 (26.3%)	
Total		102 (100%)	38 (100%)	31 (100%)	171 (100%)	
I will apply IMCI-related skills and knowledge during my practice.	Agree	90 (88.2%)	24 (63.2%)	20 (64.5%)	134 (78.4%)	NS
	Disagree	4 (3.9%)	1 (2.6%)	5 (16.1%)	10 (5.8%)	
	I don't know	8 (7.8%)	13 (34.2%)	6 (19.4%)	27 (15.8%)	
Total		102 (100%)	38 (100%)	31 (100%)	171 (100%)	
I will apply IMCI-related skills for parental counseling.	Agree	81 (79.4%)	19 (50.0%)	16 (51.6%)	116 (67.8%)	NS
	Disagree	4 (3.9%)	1 (2.6%)	2 (6.5%)	7 (4.1%)	
	I don't know	17 (16.7%)	18 (47.4%)	13 (41.9%)	48 (28.1%)	
Total		102 (100%)	38 (100%)	31 (100%)	171 (100%)	

Nearly half of the students (54.9% in phase 2, 44.7% in phase 3A, and 41.9% in phase 3B) confirmed marked improvements in their medical skills after attending the IMCI training sessions. However, 36.8% of students in phase 2 and 12.9% in phase 3 were either unaware of the clinical practice implications of the IMCI pre-service training sessions or denied that there were any benefits.

The childhood assessment benefit of the IMCI approach was acknowledged by 73.5% of patients in phase 2, 57.9% in phase 3A, and 58.1% in phase 3B of the study. Overall, nine (5.3%) students disagreed with the role of the IMCI training sessions in improving the diagnostic assessment of childhood conditions. The remaining students were unaware of the diagnostic management implications of the IMCI approach. The clinical knowledge improvement perspective of the IMCI pre-service education was appreciated by 50% of medical students. Strikingly, a similar percentage of students either disagreed or remained unaware of the correlation between clinical knowledge and IMCI training sessions.

Importantly, almost half of the medical students of phase 2 found themselves confident in dealing with sick children after the IMCI training. However, this perception was retained by only 13.2% and 37.4% of students in phase 3A and phase 3B, respectively. A similar percentage of students defied the role of IMCI training sessions in improving their confidence in managing pediatric conditions. Additionally, one-third of students revealed the positive impact of the IMCI training sessions in enhancing their counseling skills. However, the majority of the students were unaware of or disagreed with this notion.

Significant variations were obtained in the responses of students concerning the role of IMCI in improving their comprehensive clinical assessment skills of children. Agreements or unawareness or disagreements regarding this concept were registered by 39.2%, 42.7%, and 39.2% of all students, respectively.

The overall assessment of the last three responses indicated the positive perceptions of about two-thirds of students regarding the IMCI pre-service training strategy (66.1%). In addition, 67.8% and 78.4% of students aspired to utilize their IMCI-based skills in parental counseling and clinical practice, respectively. Importantly, most of the students who had more than three lectures/tutorials or practical sessions or both practical and theory sessions agreed with almost all the statements with no significant difference between the different phases. Detailed analysis is depicted in Table 4.

**Table 4:** Assessment of undergraduate medical students attending three or more IMCI tutorials/lectures/practical sessions IMCI: Integrated Management of Childhood Illness

Participants (N=25)		Educational level			Total
		Phase 2 n(%)	Phase 3A (pre-clerkship) n(%)	Phase 3B (junior clerkship) n(%)	
IMCI training/orientation is an effective teaching method.	Agree	1	7	13	21
		100.0%	100.0%	76.5%	84.0%
	Disagree	0	0	2	2
		0.0%	0.0%	11.8%	8.0%
	I don't know	0	0	2	2
		0.0%	0.0%	11.8%	8.0%
Total		1	7	17	25
		100.0%	100.0%	100.0%	100.0%
Pre-service IMCI — training has enhanced my medical skills.	Agree	1	6	12	19
		100.0%	85.7%	70.6%	76.0%
	Disagree	0	0	2	2
		0.0%	0.0%	11.8%	8.0%
	I don't know	0	1	3	4
		0.0%	14.3%	17.6%	16.0%
Total		1	7	17	25
		100.0%	100.0%	100.0%	100.0%
Pre-service IMCI — training is a practical approach for improved Assessment of childhood illnesses.	Agree	1	6	14	21
		100.0%	85.7%	82.4%	84.0%
	Disagree	0	0	2	2
		0.0%	0.0%	11.8%	8.0%
	I don't know	0	1	1	2
		0.0%	14.3%	5.9%	8.0%
Total		1	7	17	25
		100.0%	100.0%	100.0%	100.0%
Pre-service IMCI — training has improved my clinical knowledge.	Agree	1	7	11	19
		100.0%	100.0%	64.7%	76.0%
	Disagree	0	0	4	4
		0.0%	0.0%	23.5%	16.0%
	I don't know	0	0	2	2
		0.0%	0.0%	11.8%	8.0%
Total		1	7	17	25
		100.0%	100.0%	100.0%	100.0%
Because of pre-service IMCI —training, I feel confident in dealing with sick children.	Agree	1	3	11	15
		100.0%	42.9%	64.7%	60.0%
	Disagree	0	0	4	4
		0.0%	0.0%	23.5%	16.0%
	I don't know	0	4	2	6
		0.0%	57.1%	11.8%	24.0%
Total		1	7	17	25
		100.0%	100.0%	100.0%	100.0%
Pre-service IMCI — training has improved my counseling skills.	Agree	1	5	10	16
		100.0%	71.4%	58.8%	64.0%
	Disagree	0	0	3	3
		0.0%	0.0%	17.6%	12.0%
	I don’t know	0	2	4	6
		0.0%	28.6%	23.5%	24.0%
Total		1	7	17	25
		100.0%	100.0%	100.0%	100.0%
Pre-service IMCI — training has helped me to assess a child holistically.	Agree	0	5	14	19
		0.0%	71.4%	82.4%	76.0%
	Disagree	0	0	2	2
		0.0%	0.0%	11.8%	8.0%
	I don't know	1	2	1	4
		100.0%	28.6%	5.9%	16.0%
Total		1	7	17	25
		100.0%	100.0%	100.0%	100.0%
Overall, pre-service IMCI — training is an effective teaching approach.	Agree	1	3	12	16
		100.0%	42.9%	70.6%	64.0%
	Disagree	0	0	2	2
		0.0%	0.0%	11.8%	8.0%
	I don't know	0	4	3	7
		0.0%	57.1%	17.6%	28.0%
Total		1	7	17	25
		100.0%	100.0%	100.0%	100.0%
I will apply IMCI-related skills and knowledge during my practice.	Agree	1	7	14	22
		100.0%	100.0%	82.4%	88.0%
	Disagree	0	0	2	2
		0.0%	0.0%	11.8%	8.0%
	I don't know	0	0	1	1
		0.0%	0.0%	5.9%	4.0%
Total		1	7	17	25
		100.0%	100.0%	100.0%	100.0%
I will apply IMCI-related skills for parental counseling.	Agree	0	5	13	18
		0.0%	71.4%	76.5%	72.0%
	Disagree	0	0	2	2
		0.0%	0.0%	11.8%	8.0%
	I don't know	1	2	2	5
		100.0%	28.6%	11.8%	20.0%
Total		1	7	17	25
		100.0%	100.0%	100.0%	100.0%

## Discussion

Contrary to the phase 3 responses, most SQU students in phase 2 of this study responded to the questionnaire. Importantly, the current study analyzed general student perceptions regarding pre-service IMCI training, without evaluating their IMCI practical skills, knowledge, or expertise. Irrespective of their pre-service engagement in the IMCI training, the students provided positive IMCI-related perceptions. The medical students with a history of one to two IMCI tutorials/lectures responded positively to the role of the IMCI approach in improving their patient management skills and medical knowledge. Most students indicated their willingness to apply IMCI skills and knowledge in parental counseling and medical practice. In contrast, three of the questions showed less agreement with a more prominent negative perception. These are the questions that addressed the IMCI effect on students’ confidence in dealing with sick children, their counseling skills improvement, and children assessment.

The negative IMCI perceptions were attributed to several possibilities, including the limited presence of students in the IMCI lectures, inadequate training, time limitations, and high workload. Other unexplored or undefined reasons could also have impacted the IMCI training perceptions of the medical students. These gaps substantiate the requirement to improve the quality of the IMCI training sessions to improve the child illness management skills and competency of medical students. Significant differences in the responses of medical students were recorded for questions 5 and 7, respectively. These questions collected data on the confidence of students and their ability to undertake holistic assessments of pediatric populations. The non-exposure of the phase 2 students to clinical practice probably contributed to these variations in responses and the negative IMCI perceptions. Indeed, students who attended three or more IMCI sessions developed positive perceptions about their clinical practice outcomes.

The overall findings of our study concord with the outcomes of other similar studies in the literature. A recent study by Al-Araimi and Langrial (2016) revealed the capacity of the IMCI sessions to improve the patient management confidence, clinical practice skills, and clinical knowledge of medical students [19]. The study by Horwood et al. (2009) authenticated the positive perceptions of healthcare workers regarding the IMCI training sessions and their clinical practice utility [20]. Another study revealed significant outcomes regarding the role of IMCI guidelines and their clinical practice implementation in improving the medical knowledge and child management skills of medical interns [21]. Ultimately, pediatric healthcare optimization and improvement in the physician's expertise may rely on the successful implementation of the IMCI protocols in clinical settings [9,22]. On the contrary, a recent study based in Egypt confirmed the impact of improper clinical training sessions, training non-adherence, and high patient-to-physician ratio on the negative IMCI perceptions in physicians [23].

The outcomes from our study provide evidence advocating the future clinical practice implications of the IMCI training. Medical students will possibly attain substantial knowledge, skills, and best practice advantages from the IMCI training and acquire several opportunities to implement it in future clinical practice [24]. The outcomes from our study may help establish milestones to streamline IMCI training sessions and guidelines in medical schools. Despite the limited clinical practice implications of our findings, policymakers will be able to utilize them to reinforce and implement the IMCI training guidelines in pediatric settings in Oman [19].

Furthermore, by fostering digital literacy and interprofessional collaboration, we may enhance the effective utilization of artificial intelligence in the management of childhood illnesses [25]. In view of the evolving new era of healthcare, it is imperative to consistently evaluate and modify educational programs to integrate artificial intelligence technologies like ChatGPT, ensuring that the next generation of healthcare professionals is equipped to provide the highest standard of care in an increasingly digital world.

Limitations

There are several limitations to this study. First, the paper-based questionnaire restricted its access to the phase 3 students who were known to be occupied with medical school and, therefore, were difficult to reach. Second, the outcomes had a risk of recall bias, since many students without any clinical practice exposure could not recollect their previous IMCI engagements. Third, high variations in phase 2 responses among medical students restricted their comparison with phase 3 outcomes. Furthermore, a lack of consideration of the mode of learning (online versus in-person) might have impacted the knowledge and confidence level of the students toward IMCI. Future studies should accordingly utilize qualitative group discussions to collect data regarding IMCI perceptions of medical students. These group discussions will assist in minimizing ambiguity in responses and add more clarity to the overall outcomes.

## Conclusions

Overall, medical students in SQU positively perceived IMCI training implications and aspired to utilize the corresponding clinical skills and knowledge in their future practice. Approximately half of the students attending one to two training sessions believed in the positive influence of the IMCI approach on their medical knowledge and pediatric practice capacity. Most students showed a willingness to utilize IMCI skills, knowledge, and expertise in prospective parental counseling and clinical practice.

## Appendices

**Table 5:** Part 1: socio-demographic characteristics IMCI: Integrated Management of Childhood Illness, JCR: junior clerkship

Variables	Coding categories
Age	
Sex	1=male, 2=female
Educational level	Phase 2, phase 3 (pre-clerkship, phase 3 (JCR)
Duration of pre-service IMCI training received (you can choose more than one option)	One lecture/tutorial, two lectures/tutorials, ≥3 lectures/tutorials, practical sessions at JCR, family medicine rotation, practical sessions at JCR, child health rotation

**Table 6:** Part 2: perception of undergraduate medical students toward IMCI pre-service training Permission to reproduce the questionnaire has been obtained from the corresponding author of the original questionnaire [19]. IMCI: Integrated Management of Childhood Illness

	Statement	Strongly agree	Agree	I don’t know	Disagree	Strongly disagree
1	IMCI training/orientation is an effective teaching method.					
2	Pre-service IMCI — training has enhanced my medical skills.					
3	Pre-service IMCI — training is a practical approach to enhance the evaluation and diagnosis of childhood illnesses.					
4	Pre-service IMCI — training has improved my clinical knowledge.					
5	Due to pre-service IMCI —training, I feel confident in dealing with sick children.					
6	Pre-service IMCI — training has improved my counseling skills.					
7	Pre-service IMCI — training has helped me in conducting a holistic assessment of a child.					
8	All in all, pre-service IMCI — training is an effective teaching approach.					
9	I usually apply IMCI-related skills and knowledge during my practice.					
10	I usually apply IMCI-related skills for parental counseling.					
